# Membrane Biofouling Control by Surface Modification of Quaternary Ammonium Compound Using Atom-Transfer Radical-Polymerization Method with Silica Nanoparticle as Interlayer

**DOI:** 10.3390/membranes10120417

**Published:** 2020-12-11

**Authors:** Lehui Ren, Meng Ping, Xingran Zhang

**Affiliations:** State Key Laboratory of Pollution Control and Resource Reuse, Shanghai Institute of Pollution Control and Ecological Security, School of Environmental Science and Engineering, Tongji University, 1239 Siping Road, Shanghai 200092, China; 1932834@tongji.edu.cn (L.R.); pingmeng@tongji.edu.cn (M.P.)

**Keywords:** antibiofouling, polyvinylidene fluoride, silica nanoparticles, quaternary ammonium compounds, wastewater treatment

## Abstract

A facile approach to fabricate antibiofouling membrane was developed by grafting quaternary ammonium compounds (QACs) onto polyvinylidene fluoride (PVDF) membrane via surface-initiated activators regenerated by electron transfer atom-transfer radical-polymerization (ARGET ATRP) method. During the modification process, a hydrophilic silica nanoparticle layer was also immobilized onto the membrane surface as an interlayer through silicification reaction for QAC grafting, which imparted the membrane with favorable surface properties (e.g., hydrophilic and negatively charged surface). The QAC-modified membrane (MQ) showed significantly improved hydrophilicity and permeability mainly due to the introduction of silica nanoparticles and exposure of hydrophilic quaternary ammonium groups instead of long alkyl chains. Furthermore, the coverage of QAC onto membrane surface enabled MQ membrane to have clear antibacterial effect, with an inhibition rate ~99.9% of *Escherichia coli* (Gram-negative) and *Staphylococcus aureus* (Gram-positive), respectively. According to the batch filtration test, MQ had better antibiofouling performance compared to the control membrane, which was ascribed to enhanced hydrophilicity and antibacterial activity. Furthermore, the MQ membrane also exhibited impressive stability of QAC upon suffering repeated fouling–cleaning tests. The modification protocols provide a new robust way to fabricate high-performance antibiofouling QAC-based membranes for wastewater treatment.

## 1. Introduction

Membrane technology has been used extensively in water and wastewater treatment. Membrane fouling, however, remains a tough issue that severely hinders its further application [1,2]. Among all types of fouling, biofouling is particularly problematic due to its adhesive and irreversible nature [3,4,5]. Common biofouling control strategies include feed solution pretreatment [6], membrane cleaning [7,8] and membrane modification [9,10,11]. Fabrication of antibiofouling membrane has been actively pursued due to its high-efficiency and cost-saving properties.

Recently, various strategies of membrane modification have emerged for construction of antibiofouling surfaces. In general, they can be classified into passive and active methods [12]. Passive antifouling strategies aim to weaken the foulant–membrane interaction to prevent the initial adsorption of bacteria, while active strategies rely on designed surfaces that are able to inactivate the cells and thus inhibit bacterial proliferation/growth. Although they are effective in reducing the adhesion of bacteria cells to the membrane, inherent limitations to passive strategies still exist due to their inability to suppress the colonization of cells. Therefore, the latter are more attractive for their effective inhibition of microbial colonization and efficacious prevention of biofilm formation, which mainly includes incorporation of biocides into the membrane that are dependent on release-killing (e.g., silver nanoparticles [13,14,15], and metal organic frameworks containing silver and copper metals [16,17,18]) and contact-killing scenarios (e.g., quaternary ammonium compounds (QACs) [19,20,21] and carbon-based materials [22,23,24]).

QACs are well-known for developing a surface with contact-active antimicrobial features, and have thus been applied as an effective kind of antibiofouling agent in membrane modification [12,25]. Notably, for contact-mediated antibacterial strategies, intimate contact is a prerequisite for direct physical toxicity, implying that the antimicrobial efficiency depends on the exposure concentration of biocides on the membrane surface. Compared to the in situ modification method, surface grafting has the potential to provide sufficient surface coverage of antimicrobial agents to enhance antibiofouling efficiency, such as UV grafting [26], plasma grafting [27] and atom-transfer radical-polymerization (ATRP) [28]. Among these grafting methods, ATRP is attracting growing interest in the area of antifouling membrane construction since it offers a unique approach to generating a high-density antibacterial layer with controlled architecture at mild conditions. Nevertheless, the grafting of QAC with long alkyl chains often results in the decrease of membrane hydrophilicity and permeability if the concentration of QAC on the membrane surface is too high [29], since QACs are positively charged and have hydrophilic heads and hydrophobic tails. This inherent disadvantage drives us to develop QAC-modified membranes with excellent antibiofouling activity, as well as favorable intrinsic membrane properties.

In this work, we fabricated an antibiofouling polyvinylidene fluoride (PVDF) membrane by grafting QAC on membrane surface using activators regenerated by electron transfer atom-transfer radical polymerization (ARGET ATRP) method. Compared with the traditional ATRP method, ARGET-ATRP requires a smaller amount of copper catalyst and tolerate a limited amount of oxygen, which provides a convenient way of modifying the membrane surface under ordinary laboratory and industrial conditions [30,31,32]. Furthermore, as hydrophilic nanoparticles (NPs) with abundant hydroxyl groups, silica NPs were firstly introduced as an interlayer to provide active sites to the initiator that could induce homogeneous grafting of QAC via ARGET ATRP, and to improve the hydrophilicity and permeability of the modified membrane. A controlled architecture on the membrane surface was constructed: (i) polydopamine (PDA)/polyethylenimine (PEI) was initially coated on membrane surface to provide abundant positively charged functional groups; (ii) a hydrophilic silica nanoparticle layer was synthesized onto positively charged surfaces through silicification reaction; (iii) 2-bromoisobutyryl bromide (BiBB) was then decorated onto the silica layer; and finally (iv) QAC polymerization was induced for producing an QAC-based antibacterial surface via ARGET ATRP. The incorporation of the silica NPs layer not only provides hydrophilicity to neutralize the negative effects by QAC (hydrophobicity) on membrane properties, but also brings about stable and lasting antibiofouling activities by the covalent bonding with QAC. Additionally, the exposure of positively charged quaternary ammonium moieties (hydrophilic heads of QAC) could impart membranes with antibacterial efficiency, as well as enhanced hydrophilicity. The modification protocols provide a new robust way to fabricate high-performance antibiofouling QAC-based membranes for wastewater treatment.

## 2. Materials and methods

### 2.1. Reagents

Base polyvinylidene fluoride (PVDF) microfiltration membrane with a pore size 0.22 µm was purchased from Millipore. Tris (hydroxymethyl) aminomethane, dopamine hydrochloride, and polyethylenimine (PEI) with an M_w_ = 600 Da were provided by Sigma-Aldrich (St. Louis, MO, USA) for fabricating polydopamine (PDA)/PEI-modified membranes. Tris-buffer solution and tetramethyl orthosilicate (TMOS) were purchased from TCI (TCI Development Co., Ltd., Shanghai, China). Triethylamine (TEA) (>99%), tris(2-pyridylmethyl)amine (TPMA), α-bromoisobutyryl bromide (BiBB) (98%), L-ascorbic acid and copper (II) chloride were received from Sigma-Aldrich for preparing QAC-decorated membranes. A kind of QAC, [2-(Acryloyloxy)ethyl]trimethylammonium chloride solution (DAC) (Sigma-Aldrich), was used to create an antibacterial layer. *Staphylococcus aureus* (*S. aureus*, ATCC6538) and *Escherichia coli* (*E. coli*, ATCC25922), representing Gram-positive and Gram-negative strains, respectively, were purchased from Shanghai Weike and used as model bacteria. All chemicals used were of analytical grade unless specified otherwise.

### 2.2. Membrane Fabrication

**Figure 1:**
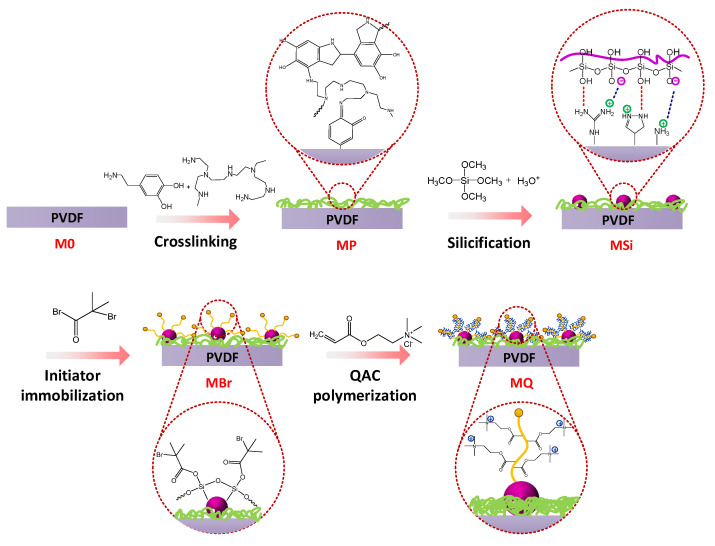
Schematic of modification procedures using ATRP and silica nanoparticles as interlayer for grafting QAC onto membrane surfaces for mitigating biofouling.

Figure 1 shows the fabrication process of QAC-modified membranes. PDA/PEI layer was initially coated on the pristine PVDF membrane. In brief, a membrane coupon (M0) was immersed into 200 mL tris-buffer solution (dissolving 2 mg/L dopamine hydrochloride and PEI, 1:1 of mass ratio). Dopamine can be oxidized under alkaline conditions and then self-aggregated to form a cross-linked layer [33]. Additionally, PEI can connect with dopamine via reaction between catechol and amino group, providing more positively charged functional groups in favor of following immobilization of SiO_2_ NPs [34,35]. After deposition of 4 h, the resulting PDA/PEI-modified membrane (MP) was washed by deionized water overnight to remove the residuals.

Secondly, TMOS was nucleated on the surface of MP via silicification reaction by electrostatic interaction. Briefly, TMOS was added into 1 mM HCl solution (15:1000 of volume ratio) under stirring (200 rpm) for 15 min, and then diluted by 0.2 M phosphate buffer solution (100 mL). The MP membrane was then immersed in this solution under stirring (200 rpm) for 6 h at 25 °C. Ultimately, the silica-decorated membrane (MSi) was washed overnight to remove impurities.

Thirdly, α-bromoisobutyryl bromide (BiBB), which was the ATRP initiator, was immobilized onto MSi in nitrogen gas atmosphere. The MSi membrane was immersed into a mixture of triethylamine (2 mL) and dichloromethane (100 mL). BiBB (2 mL) was then dosed into the stirred solution at the temperature of 25 °C after N_2_ degassing for 15 min. After 12 h reaction, the BiBB-immobilized membrane (MBr) was subject to sequential washing by dichloromethane, ethanol and deionized water. The successful coating of the BiBB layer was confirmed by a yellow color that appeared on the membrane surface (Supplementary Materials Figure S1) [30].

Finally, the ARGET-ATRP process was initiated to graft QAC onto BiBB-decorated membrane through three steps: (i) The reduction of Cu (II) to Cu (I) occurred with ascorbic acid being as reducing agent; (ii) active radicals could be generated by reaction between complex Cu(I)-TPMA and CBr bonds; and (iii) DAC polymerization was then induced by the radicals to form antibacterial layer. In brief, the MBr membrane was immersed into isopropanol/water solution (100 mL, 1:1 of volume ratio) in which DAC (28 mmol) was dissolved in an opaque bottle. Prior to degassing with N_2_, another isopropanol aqueous solution (4 mL) containing CuCl_2_ (14.8 μmol) and TPMA (96.4 μmol) was injected into the bottle. After N_2_ degassing, ascorbic acid (2.2 mmol in 4 mL in 50 vol.% isopropanol/water solution) was added to induce polymerization (24 h of reaction time). The resulting QAC-grafted membrane (MQ) was subjected to washing by deionized water to remove residuals. Relative information of all the membranes is shown in Table 1.

### 2.3. Membrane Characterization

The chemical compositions of membrane surface were determined by X-Ray photoelectron spectroscopy (XPS, AXIS UltraDLD, Kratos, UK) and attenuated total reflection flourier transformed infrared spectroscopy (ATR-FTIR, Nicolet 5700, Thermo, USA). The structure and morphology of membranes were observed by scanning electron microscope (SEM, SU8010, Hitachi, Japan). The charge of membranes was determined by Anton-Paar streaming potential analyzer (EKA 1.00) [36]. A capillary flow porometer (Proplux 100, Porometer, Belgium) was used to determine membrane pore sizes. Sessile drop method was employed to determine membrane surface hydrophilicity using water [37]. A dead-end filtration cell at 10 kPa was used to measure the water permeability of membranes for three times. Membrane rejection behaviors were also determined, with the procedures documented in Supplementary Materials Section S1.

### 2.4. Evaluation of Antibacterial Activity

Model Gram-negative and Gram-positive bacteria, *E. coli* and *S. aureus*, were used to determine antibacterial performance of membranes according to the method in the literature [9,38,39]. After ultraviolet (UV) sterilization for 30 min, 0.5 cm^2^ membranes were immersed in 1 mL bacterial suspension with 108 cells/mL (37 °C) for 3 h (the bacterial suspension without membranes was taken as control samples). Subsequently, the membranes were rinsed thrice with 0.01 M sterile phosphate buffered saline (PBS, pH = 7.4) and then placed into tubes containing fresh PBS solution (10 mL). The attached bacteria onto membrane surface were removed by ultrasonication for 10 min. The cell suspension was diluted in serial and then incubated on LB plates at 37 °C overnight. The viable cells on the membranes were characterized by counting colonies in term of Colony-Forming Units (CFU). Visualization of bacterial cells adhered to the membrane surfaces after 3 h contact was performed using SEM as described previously [40].

### 2.5. Evaluation of Antibiofouling Performance

A dead-end filtration cell was used to test the antibiofouling performance of membranes (38.5 cm^2^ of effective membrane area) at room temperature. The synthetic municipal wastewater (600 mg/L CH_3_COOH, 22 mg/L KH_2_PO_4_, 130 mg/L NH_4_Cl, 412 mg/L MgSO_4_, 11.5 mg/L CaCl_2_, and 1 g/L bovine albumin (BSA)) with *S. aureus* (10^7^ cells/mL) was used as artificial wastewater (pH = 7) [41]. The membrane coupons were operated in the cell at 20 kPa for 5 h and then removed for further characterization. The foulant (biofilm) structure on biofouled membrane surfaces was analyzed by confocal laser scanning microscopy (CLSM, Nikon A1, Nikon, Japan). Concavalin A (Con A), SYPRO Orange Protein Gel Stain (Molecular Probes, Eugene, OR, USA) and LIVE/DEAD Cell Kits were used to label polysaccharide, protein and viable/dead cells on the bio-fouled membrane coupons (1 cm^2^), respectively, which was then subject to CLSM observation.

### 2.6. Stability Evaluation of the QAC-Modified Membrane

Repeated fouling and cleaning tests were performed to evaluate QAC stability [42]. Briefly, MQ samples (each with an effective surface area 0.5 cm^2^) were incubated with 1 mL *E. coli* or *S. aureus* bacteria suspensions containing 10^7^ cells/mL for 3 h. Subsequently, 0.5 vol.‰ NaClO/water solution was used to clean the bio-fouled membranes for 2 h. Biofouling–cleaning tests were performed for three cycles in total. After each cycle, PBS solution was used to soak the membrane coupons for removing residual NaClO, and antibacterial activity of membranes was then evaluated as descried in Section 2.4.

## 3. Results and Discussion

### 3.1. Membrane Characterization

**Figure 2:**
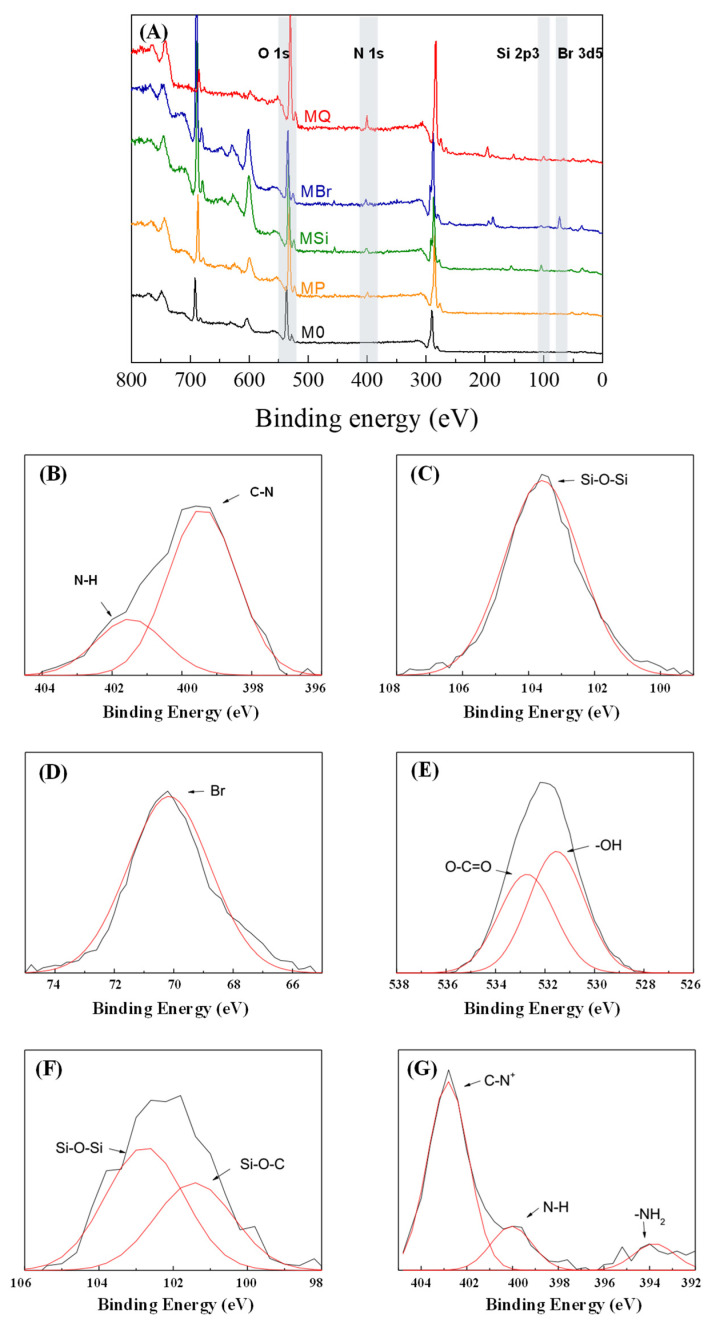
(**A**) XPS spectra. High-resolution XPS spectra of (**B**) N 1s of MP, (**C**) Si 2p3 of MSi, (**D**) Br 3d5 of MBr, (**E**) O 1s of MBr, (**F**) Si 2p3 of MBr and (**G**) N 1s of MQ.

The chemical compositions of the membrane surface were analyzed by XPS. The results of the XPS full spectrum and its relative elements distribution confirmed the successful stepwise modification of QAC-based membrane (Figure 2A and Table 2). Only four elements (carbon (C), oxygen (O), fluorine (F) and nitrogen (N)) were observed from pristine membrane and PDA/PEI coated membrane. However, the concentration of nitrogen increased after the deposition of PDA/PEI. It can be clearly seen from Figure 2B that the peak of N 1s XPS spectra is divided by two peaks, which corresponds to N-H [43] and C-N at binding energies of 401.3 eV and 399.3 eV, respectively, indicating the existence of PDA/PEI on the membrane surface. After silicification reaction, element of silicon appears for MSi (Figure 2A and Table 2), suggesting the existence of the silica layer (Figure 2C). However, the grafting of bromine onto MSi surface shielded the intermediate silica layer, leading to a lower peak value (Figure 2A) and surface concentration of Si (0.93% in Table 2). Meanwhile, a peak at 70.3 eV for Br 3d5 was observed in XPS spectra (Figure 2D), and about 1.33% bromine on MBr membrane surface was reached with approximately 2.47% of the surface weight ratio of BiBB to MSi. Additionally, two resolved peaks at 531.4 eV and 532.8 eV associated with O-H and O–C=O in O 1s peak, and the peak (Figure 2E) at 101.4 eV representing Si-O-C in Si 2p3 (Figure 2F), correspond to the chemical structure of BiBB grafted on the surface of MBr membrane [44]. After immobilization of QAC, the presence of nitrogen (i.e., NR_4_^+^) partially covered up bromine (i.e., C–Br), resulting in higher concentration of nitrogen and lower concentration of bromine on the MQ membrane surface compared to that on MBr (Table 2). Furthermore, the dominant peak was observed at 402.8 eV (C-N^+^ peak) [29], suggesting the successful grafting of DAC onto the membrane surface (Figure 2G).

**Figure 3:**
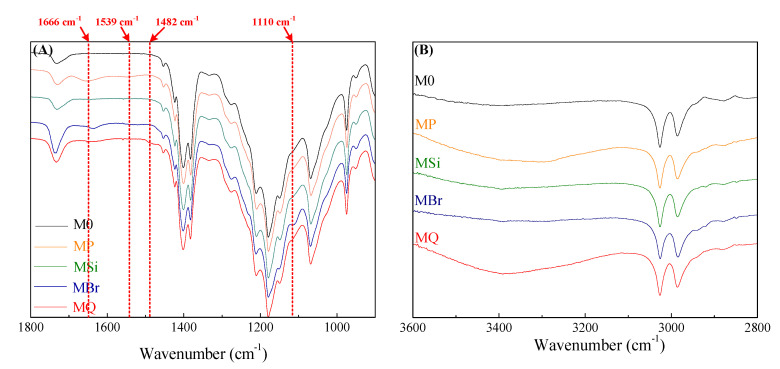
ATR-FTIR spectra from (**A**) 1800 cm^−1^ to 900 cm^−1^ and (**B**) from 2800 cm^−1^ to 3600 cm^−1^ of the pristine and modified membranes.

ATR-FTIR spectra (Figure 3 A,B) of membranes (i.e., M0, MP, MSi, MBr and MQ) also confirmed the successful stepwise modification of DAC. Absorption peaks at 1539 cm^−1^ and 1666 cm^−1^ were observed in MP, which are attributed to the vibrations of the amide groups (C-N-H and N-C-O) from PDA/PEI layer [30], respectively (Figure 3A). Additionally, the peak near 1110 cm^−1^ is associated with Si-O-C groups from MSi [29], suggesting that the silica nanoparticle interlayer has been incorporated in the modification process (Figure 3A). The intensity changes of hydroxyl peaks around 3500 cm^−1^ for the MSi and MBr membranes demonstrated the successful reaction between BiBB and silica nanoparticles (Figure 3B). Compared to MSi, the reduced intensity of hydroxyl peak of MBr revealed that a part of the hydroxyl groups of silica had reacted with bromooctanoyl group of BiBB, suggesting successful grafting of BiBB. After DAC grafting, the peak at 1482 cm^−1^ was intensified in the spectra of MQ due to the presence of quaternary ammonium groups [29]. Furthermore, the coverage of hydrophilic brushes of QAC would induce the attraction of water, thus resulting in increased intensity of hydroxyl peak of MQ (Figure 3B).

**Figure 4:**
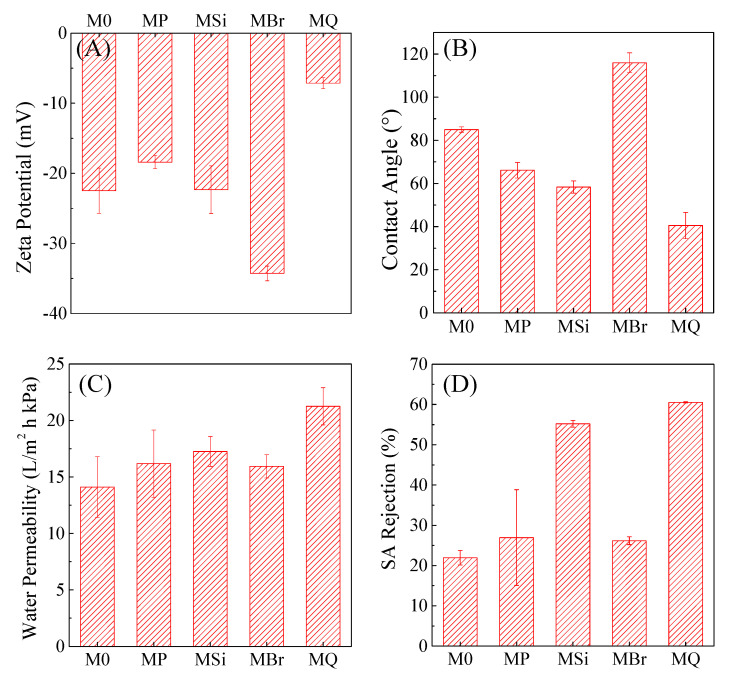
Characterization of membrane properties. (**A**) Zeta potential, (**B**) Contact angle of DI water, (**C**) Water permeability (10 kPa), (**D**) SA rejection. The number of measurements: *n* = 3 for zeta potential, water permeability and SA rejection; *n* = 7 for contact angle.

Figure 4A shows that the Zeta potential of MP (i.e., PDA/PEI-coated membrane) was less negatively charged (−18.4 ± 0.9 mV for MP and −22.5 ± 3.2 mV for M0). Silica acid is prone to adsorbing to the less negative-charged surface, which is beneficial to the growth of silica nanoparticles. The immobilization of silica nanoparticles led to the change surface charge to −22.3 ± 3.4 mV for MSi. QAC immobilization resulted in the shift of membrane surface charge to −7.1 ± 0.8 mV, ascribed to the coverage of the positively charged QAC on the membrane surface [45,46]. Furthermore, the charge of membrane did not affect the polymerization of QAC. Although zeta potential of BiBB-modified membrane (MBr) was lower than that of MSi, the QAC monomers preferred to polymerize via ARGET ATRP rather than adsorb onto membrane surface.

Figure 4B shows the change of contact angle during the stepwise modification process. After PDA/PEI coating, membrane hydrophilicity was enhanced due to the amino groups of PEI (Figure 4B). Silica decoration provides more hydrophilic groups leading to further decrease of contact angle. The MQ membrane had the lowest surface contact angle (e.g., the strongest hydrophilicity) (Figure 4B), attributed to the exposure of hydrophilic QAC heads (nitrogen atoms) instead of long alkyl chains and the contribution of silica nanoparticle interlayer. Membrane permeability for M0, MP, MSi, MBr and MQ had a similar changing trend (Figure 4C). MQ had a higher water permeability than M0, while no significant difference was observed among M0 and other modified membranes. Furthermore, MSi showed higher hydrophilicity and permeability than MP, suggesting the silica played a critical role for the improvement of membrane properties. The above-mentioned results also demonstrated that the immobilized silica nanoparticle layer imparted the membrane (MSi) with favorable surface properties, i.e., more hydrophilic, increased negative charge potential and thus enhanced water permeability compared to M0.

Surface and cross-section morphologies did not show difference among M0 and modified membranes (Supplementary Materials Figure S2). Also, there were no significant differences (*p* > 0.05) in pore size among M0 and other types of membranes (Supplementary Materials Figure S3), suggesting that the modification procedures did not have negative impact on membrane surface and matrix morphologies. The silica nanoparticles are combined with the inherent microporous structure of the membrane surface, which is responsible for the increased hydrophilicity and permeability yet with negligible change in pore size. However, SA rejection rate increased significantly after silica decoration (Figure 4D), which might be attributed to enhanced repulsive interaction between hydrophobic SA molecular and hydrophilic silica nanoparticles. In contrast, SA rejection decreased after grafting BiBB onto membrane surfaces, mainly due to the increased intrinsic hydrophobic property of the MBr membrane. Moreover, SA rejection of MQ (Figure 4D) was the highest among the membranes, possibly because the coverage of hydrophilic head of QAC could induce the attraction of water, thus resulting in the swelling of polymers and the enhancement of SA rejection.

### 3.2. Evaluation of Antibacterial Activity

**Figure 5:**
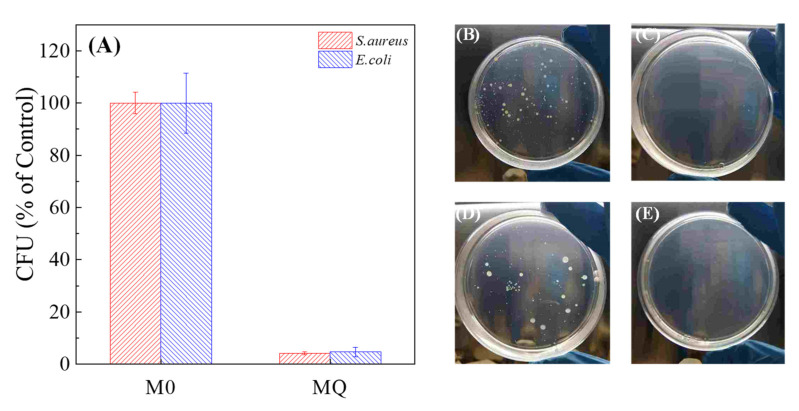
(**A**) Percentage of CFU on M0 and MQ after exposure to bacterial suspensions containing *S. aureus* and *E. coli* for 3 h. Photos displaying the number of colonies of *S. aureus* on (**B**) M0 and (**C**) MQ membrane, *E. coli* on (**D**) M0 and (**E**) MQ membrane after contact for 3 h.

The antibacterial activity of QAC-modified membrane (MQ) was evaluated by CFU counting method by immersing the membrane samples into bacteria suspensions for 3 h (*E. coli* or *S. aureus* suspension). The attached bacteria on the membrane surface were collected and incubated with LB agar plats, with CFU counted. Figure 5A,B exhibit a significant decrease of CFU on MQ membranes compared with M0 membranes. MQ membranes showed an inhibition rate of ~99.9% (in terms of CFU decrease) for both *E. coli* and *S. aureus*, demonstrating that the introduction of QAC on membrane surface by ATRP was effective for suppressing biofouling.

**Figure 6:**
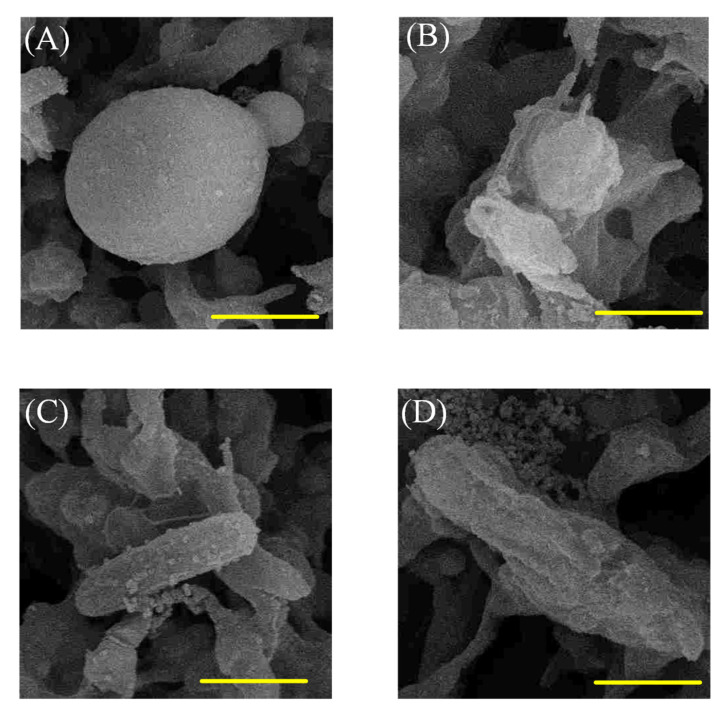
SEM images displaying *S. aureus* on (**A**) M0 and (**B**) MQ, and *E. coli* on (**C**) M0 and (**D**) MQ membranes. The scale of yellow bar is 1 µm.

SEM images exhibit the morphologies of the deposited bacterial cells on M0 and MQ membranes (Figure 6). Severe damage to both *S. aureus* and *E. coli* cells on MQ membrane occurred, which is consistent with the results of CFU tests. In contrast, no obvious damage to the adhered cells on the M0 surface was observed. These results further confirmed that QAC decorated membrane (MQ) had impressive antimicrobial activity.

Currently, there are two mechanisms of bacterial killing for QAC-based surfaces: one assumes that cell membrane can be damaged by penetration of long chains of QAC grafted on the surface [47], and the other is based on electrical interaction between cell membranes and QAC-grafted surfaces (i.e., exchange of cationic ions on cell membranes and positively charged surfaces [48] or selective adhesion of negatively charged phospholipids in the cell membrane onto the cationic surface [49]). The mechanisms imply that exposure of either tails or heads of QAC could impart the membranes with antibacterial properties. However, it is taken for granted that the coverage of long alkyl chains of QAC (i.e., tails) results in the decrease of membrane hydrophilicity and permeability. In this study, the introduction of quaternary ammonium moieties with QAC heads towards outside the surface can also impart the membranes with antimicrobial properties via electrical interaction as well as avoiding the increase of hydrophobicity by long alkyl chains.

### 3.3. Antibiofouling Performance

**Figure 7:**
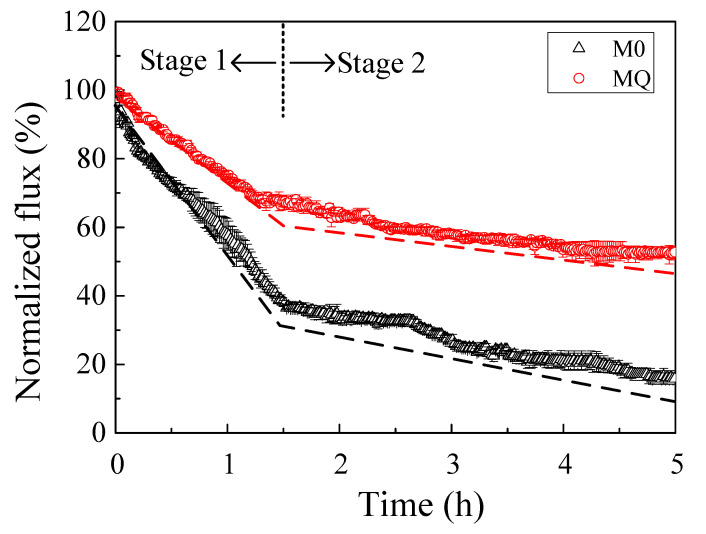
Normalized water flux of M0 and MQ due to biofouling by *S. aureus*. The initial bacterial concentration was 10^7^ cells/mL. The dead-end filtration cell was operated at 10 kPa (*n* = 3). The black and red dash lines represent decline tendency of M0 and MQ membranes.

Antibiofouling behaviors of QAC-grafted membrane (MQ) were further determined in a dead-end filtration cell using synthetic wastewater containing 10^7^ cells/mL (*S. aureus*) as a feed solution. During the experiment, the decrease of water flux was attributed to the adhesion of bacteria and growth of biofilm on the membrane surface. Figure 7 shows the changes of normalized water flux during the 5 h filtration process. MQ exhibited impressive antibiofouling ability, with ~50% flux decline compared to ~80% decline of M0. Moreover, the normalized water flux profile in both membranes exhibited two-stage phenomenon, i.e., a rapid decrease stage (stage 1) followed by a slow one (stage 2). The sharp water flux decrease stage (stage 1) of MQ was milder than that of M0, although similar water flux decrease rates were observed at stage 2 for the two membranes. A similar phenomenon was found in an MBR system operating QAC-modified membranes [50,51]. This indicates that QAC can cause contact-mediated toxicity towards bacterial cells to effectively reduce bacteria attachment/growth on membranes. This is mainly attributed to cationic adsorption of positively charged quaternary ammonium moieties, inducing damage to its function such as cell signaling and transportation of substance in and out of cell membrane [52,53]. In addition, the enhanced hydrophilicity attributed to the grafting of MQ (Figure 4D) might provide anti-adhesive properties towards bacteria, thus inhibiting subsequent formation of biofilm.

**Figure 8:**
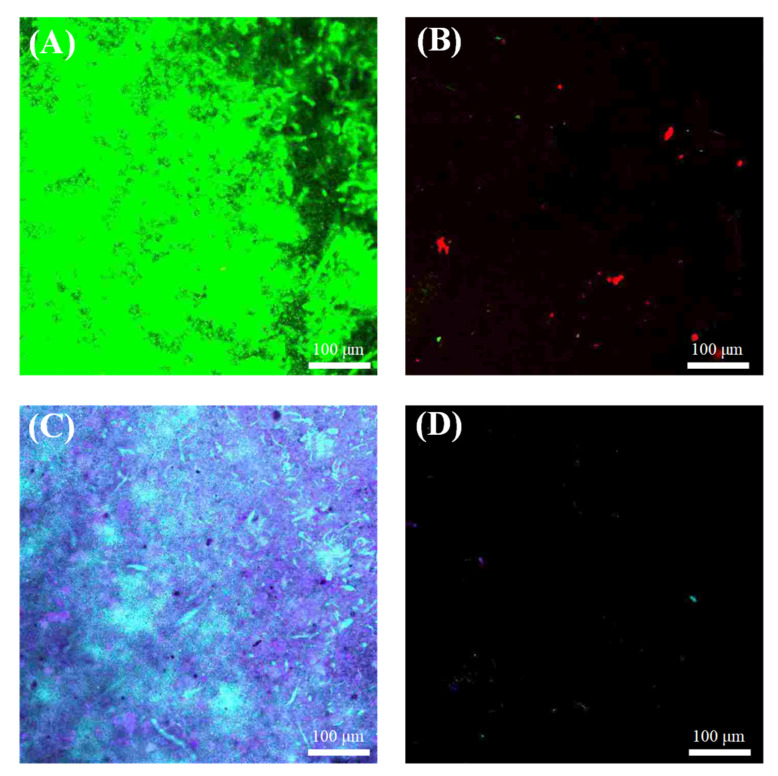
CLSM images exhibiting the biofilms on M0 ((**A**) and (**C**)) and MQ ((**B**) and (**D**)) at the end of filtration. “Live”, “dead” cells, α-polysaccharides and proteins in biofilms were labeled by SYTO 9 (green), PI (red), Con A (blue) and SYPRO Orange (yellow), respectively.

CLSM analysis was further used to investigate the biofilm structure of the fouled membrane for understanding the role of QAC in antibiofouling behaviors. As shown in Figure 8A,B, it is evident that the live cells on MQ were less than those on M0 membrane. In contrast, the dead cells on MQ were more than those on M0. Furthermore, Figure 8C,D show that the quantity of α-polysaccharides and proteins on MQ was also less than that on M0 membrane. Three-dimension images (Supplementary Materials Figure S4) further confirmed that a thinner biofouling layer was formed on the MQ membrane compared to M0, suggesting a lower biovolume on MQ. The decreased accumulation of biofoulants and inhibited growth of biofilm are in accordance with the antibacterial activity results as shown in Figure 5 and Figure 6.

The antibiofouling behaviors of MQ might be attributed to (i) enhanced hydrophilicity imparted by the coverage of silica NPs and quaternary ammonium groups enabling the membrane to mitigate the adhesion of bacteria and also their consequent secretion of microbial products (polysaccharides and proteins) on membrane surfaces, and (ii) destabilization and/or loss of natural substance of bacteria through contact with quaternary ammonium groups resulting in cell death and thus inhibition of biofilm formation. Positively charged quaternary ammonium groups could replace divalent cations (i.e., Mg^2+^ and Ca^2+^) on the cell membrane [52,53] or attract negatively charged phospholipids onto the cationic surface [49], leading to cell destabilization and causing damage to integrity. Furthermore, the damaged cells have possibility to cause negative feedback on the process of metabolism via cell regulation or signaling, which can possibly trigger an SOS response of bacteria close to the membrane surface and thus inhibit biofilm growth [11,54,55].

### 3.4. Evaluation of QAC Stability by Repeated Fouling and Cleaning Procedures

**Figure 9:**
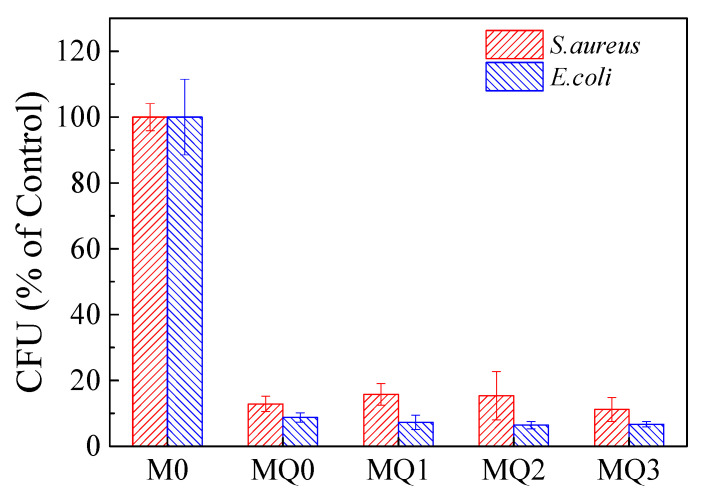
CFU (percentage of control) on M0 and MQ relative to that on M0 suffered from three fouling–cleaning cycles. A cycle includes exposure to bacterial suspension for 3 h followed by 2 h cleaning by 0.5 vol.‰ NaClO. The 0, 1st, 2nd and 3rd fouling–cleaning cycle is denoted as MQ-0, MQ-1, MQ-2 and MQ-3, respectively (*n* = 3).

Stability of QAC is of great significance for antibiofouling efficiency of membranes during long-term operation. The mechanical damage from bacteria might be negligible due to short incubation time. Figure 9 shows the changes of antibacterial efficiencies when M0 and MQ suffered from three fouling and cleaning cycles. After the third fouling–cleaning cycle (i.e., in total 9 h contacting and 6 h cleaning), the MQ membrane exhibited inhibition rates of ~88.8% against *S. aureus* and ~93.3% against *E. coli*, suggesting that chemical cleaning by NaClO did not significantly affect the antibacterial efficiency of MQ. The results imply that MQ membrane may have a strong potential for real wastewater treatment owing to the lasting antibiofouling ability, which are still needed to be demonstrated in future study.

## 4. Conclusions

In this study, a novel approach to membrane modification was developed to mitigate membrane biofouling as well as enhance membrane intrinsic properties. A hydrophilic silica nanoparticle layer was immobilized onto the membrane surface as an interlayer through silicification reaction for QAC grafting, which imparted the membrane with favorable surface properties (e.g., hydrophilic and negatively charged surface). Using surface-initiated ARGET ATRP method, QAC was then successfully grafted onto PVDF membrane (MQ). The MQ membrane had significantly improved hydrophilicity and permeability mainly due to introduction of silica nanoparticles and the exposure of hydrophilic quaternary ammonium groups instead of long alkyl chains. The CFU counting method and SEM images demonstrated that MQ had an inhibition rate of ~99.9% against both *E. coli* and *S. aureus*, respectively, by destruction of cell integrity. Batch tests also showed that MQ had better antibiofouling performance compared to M0, which was attributed to its enhanced hydrophilicity and antibacterial activity. The MQ membrane also had favorable stability in repeated fouling–cleaning tests, demonstrating a strong potential for real wastewater treatment. This study mainly focused on developing an approach to fabricating QAC-based membrane with both antibiofouling capability and high membrane performance, and silica nanoparticles were thus introduced as an interlayer to immobilize QAC as well as to impart the membrane with favorable surface properties. Further study needs to optimize membrane preparation procedures, such as deposition time of interlayer and the exposure concentration of QAC, to achieve better membrane performance.

## Figures and Tables

**Table 1:** Relative information of all the membranes.

No.	Compositions	Remarks
M0	The base MF membrane	From Millipore
MP	M0 coated with a PDA/PEI layer	By crosslinking
MSi	MP decorated with silica NPs layer	Via silicification reaction
MBr	MSi grafted with BiBB	BiBB as an initiator
MQ	MBr grafted with QACs	Via ARGET-ATRP

**Table 2:** Membrane surface compositions analyzed by XPS.

No.	Surface element Composition (%)					
	C	F	O	N	Si	Br
M0	61.92	13.05	24.71	0.31		
MP	63.57	11.03	22.98	2.42		
MSi	54.21	22.92	18.51	1.55	2.81	
MBr	57.14	23.3	15.62	1.68	0.93	1.33
MQ	65.64	5.56	22.97	3.45	1.98	0.4

## Supplementary Materials

The following are available online at https://www.mdpi.com/2077-0375/10/12/417/s1, Section S1: Membrane rejection behaviors; Figure S1: The images of M0, MP, MSi, MBr and MQ; Figure S2: the images of M0, MP, MSi, MBr and MQ; Figure S3: The images of M0, MP, MSi, MBr and MQ; Figure S4: CLSM 3D images of M0 and MQ membranes.

## References

[1] Fane A.G., Wang R., Hu M. (2015). Synthetic Membranes for Water Purification: Status and Future, Angew. Chem. Int. Edit..

[2] Jiang S., Li Y., Ladewig B.P. (2017). A review of reverse osmosis membrane fouling and control strategies. Sci. Total Environ..

[3] Lu H., Xue Z., Saikaly P., Nunes S.P., Bluver T.R., Liu W. (2016). Membrane biofouling in a wastewater nitrification reactor: Microbial succession from autotrophic colonization to heterotrophic domination. Water Res..

[4] She Q., Wang R., Fane A.G., Tang C.Y. (2016). Membrane fouling in osmotically driven membrane processes: A review. J. Membr. Sci..

[5] Meng F., Zhang S.Q., Oh Y., Zhou Z., Shin H., Chae S. (2017). Fouling in membrane bioreactors: An updated review. Water Res..

[6] Lutchmiah K., Verliefde A.R., Roest K., Rietveld L.C., Cornelissen E.R. (2014). Forward osmosis for application in wastewater treatment: A review. Water Res..

[7] Xu H., Liu Y. (2011). Control and cleaning of membrane biofouling by energy uncoupling and cellular communication. Environ. Sci. Technol..

[8] Han X., Wang Z., Wang X., Zheng X., Ma J., Wu Z. (2016). Microbial responses to membrane cleaning using sodium hypochlorite in membrane bioreactors: Cell integrity, key enzymes and intracellular reactive oxygen species. Water Res..

[9] Liu C., Faria A.F., Ma J., Elimelech M. (2017). Mitigation of Biofilm Development on Thin-Film Composite Membranes Functionalized with Zwitterionic Polymers and Silver Nanoparticles. Environ. Sci. Technol..

[10] Qi L., Hu Y., Liu Z., An X., Zeev E.B. (2018). Improved anti-biofouling performance of thin-film composite forward-osmosis membranes containing passive and active moieties. Environ. Sci. Technol..

[11] Zhang X., Ma J., Tang C.Y., Wang Z., Ng H.Y., Wu Z. (2016). Antibiofouling polyvinylidene fluoride membrane modified by quaternary ammonium compound: Direct contact-killing versus induced indirect contact-killing. Environ. Sci. Technol..

[12] Zhang R., Liu Y., He M., Su Y., Zhao X., Elimelech M., Jiang Z. (2016). Antifouling membranes for sustainable water purification: Strategies and mechanisms. Chem. Soc. Rev..

[13] Yang Z., Wu Y., Wang J., Cao B., Tang C.Y. (2016). In situ reduction of silver by polydopamine: A novel antimicrobial modification of a thin-film composite polyamide membrane. Environ. Sci. Technol..

[14] Wu J., Yu C., Li Q. (2017). Novel regenerable antimicrobial nanocomposite membranes: Effect of silver loading and valence state. J. Membr. Sci..

[15] Zhang X., Ping M., Wu Z., Tang C.Y., Wang Z. (2020). Microfiltration membranes modified by silver-decorated biomimetic silica nanopollens for mitigating biofouling: Synergetic effects of nanopollens and silver nanoparticles. J. Membr. Sci..

[16] Wen Y., Chen Y., Wu Z., Liu M., Wang Z. (2019). Thin-film nanocomposite membranes incorporated with water stable metal-organic framework CuBTTri for mitigating biofouling. J. Membr. Sci..

[17] Seyedpour S.F., Rahimpour A., Najafpour G. (2018). Facile in-situ assembly of silver-based MOFs to surface functionalization of TFC membrane: A novel approach toward long-lasting biofouling mitigation. J. Membr. Sci..

[18] Mozafari M., Seyedpour S.F., Salestan S.K., Rahimpour A., Shamsabadi A.A., Firouzjaei M.D., Esfahani M.R., Tiraferri A., Mohsenian H., Sangermano M. (2019). Facile Cu-BTC surface modification of thin chitosan film coated polyethersulfone membranes with improved antifouling properties for sustainable removal of manganese. J. Membr. Sci..

[19] Zhu J., Hou J., Zhang Y., Tian M., He T., Liu J., Chen V. (2018). Polymeric antimicrobial membranes enabled by nanomaterials for water treatment. J. Membr. Sci..

[20] Ping M., Zhang X., Liu M., Wu Z., Wang Z. (2018). Surface modification of polyvinylidene fluoride membrane by atom-transfer radical-polymerization of quaternary ammonium compound for mitigating biofouling. J. Membr. Sci..

[21] Lee S., Cho H., Ha Y., Kim S., Chung B., Son W., Kang K., Jung Y., Park K., Lee J. (2017). Enhancing the durability of filtration the ultrafine aerosol by electrospun polymer filter containing quaternary ammonium moiety. Polymer.

[22] Perreault F., Jaramillo H., Xie M., Ude M., Nghiem L.D., Elimelech M. (2016). Biofouling mitigation in forward osmosis using graphene oxide functionalized thin-film composite membranes. Environ. Sci. Technol..

[23] Sun M., Boo C., Shi W., Rolf J., Shaulsky E., Cheng W., Plata D.L., Qu J., Elimelech M. (2019). Engineering Carbon Nanotube Forest Superstructure for Robust Thermal Desalination Membranes. Adv. Funct. Mater..

[24] Yousefi N., Lu X., Elimelech M., Tufenkji N. (2019). Environmental performance of graphene-based 3D macrostructures. Nat. Nanotechnol..

[25] Miller D., Dreyer D., Bielawski C., Paul D., Freeman B. (2017). Surface modification of water purification membranes: A review. Angew. Chem. Int. Ed..

[26] Galiano F., André Schmidt S., Ye X., Kumar R., Mancuso R., Curcio E., Gabriele B., Hoinkis J., Figoli A. (2018). UV-LED induced bicontinuous microemulsions polymerization for surface modification of commercial membranes-enhancing the antifouling properties. Sep. Purif. Technol..

[27] Wang W., Zheng Z., Huang X., Fan W., Yu W., Zhang Z., Li L., Mao C. (2017). Hemocompatibility and oxygenation performance of polysulfone membranes grafted with polyethylene glycol and heparin by plasma-induced surface modification. J. Biomed. Mater. Res. B.

[28] Yue W., Li H., Xiang T., Qin H., Sun S., Zhao C. (2013). Grafting of zwitterion from polysulfone membrane via surface-initiated ATRP with enhanced antifouling property and biocompatibility. J. Membr. Sci..

[29] Meng J., Zhang X., Ni L., Tang Z., Zhang Y. (2015). Antibacterial cellulose membrane via one-step covalent immobilization of ammonium/amine groups. Desalination.

[30] Ye G., Lee J., Perreault F., Elimelech M. (2015). Controlled architecture of dual-functional block copolymer brushes on thin-film composite membranes for integrated “Defending” and “Attacking” strategies against biofouling. ACS Appl. Mater. Interfaces.

[31] Carter B., Sengupta A., Qian X., Ulbricht M., Wichramasinghe S. (2018). Controlling external versus internal pore modification of ultrafiltration membranes using surface-initiated AGET-ATRP. J. Membr. Sci..

[32] Keating J., Sorci M., Kocsis I., Setaro A., Barboiu M., Underhill P., Belfort G. (2018). Atmospheric pressure plasma—ARGET ATRP modification of poly(ether sulfone) membranes: A combination attack. J. Membr. Sci..

[33] Xue Q., Cao H., Meng F., Quan M., Gong Y.K. (2017). Cell membrane mimetic coating immobilized by mussel-inspired adhesion on commercial ultrafiltration membrane to enhance antifouling performance. J. Membr. Sci..

[34] Yang H.C., Liao K.J., Huang H., Wu Q.Y., Wan L.S. (2014). Mussel-inspired modification of a polymer membrane for ultra-high water permeability and oil-in-water emulsion separation. J. Mater. Chem. A.

[35] Li M., Xu J., Chang C.Y., Feng C., Zhang L. (2014). Bioinspired fabrication of composite nanofiltration membrane based on the formation of DA/PEI layer followed by crosslinking. J. Membr. Sci..

[36] Zhang J., Xu Z., Shan M., Zhou B., Li Y., Li B., Niu J., Qian X. (2013). Synergetic effects of oxidized carbon nanotubes and graphene oxide on fouling control and anti-fouling mechanism of polyvinylidene fluoride ultrafiltration membranes. J. Membr. Sci..

[37] Wang Q., Wang Z., Wu Z. (2012). Effects of solvent compositions on physicochemical properties and anti-fouling ability of PVDF microfiltration membranes for wastewater treatment. Desalination.

[38] Liu C., Lee J., Ma J., Elimelech M. (2017). Antifouling thin-film composite membranes by controlled architecture of zwitterionic polymer brush layer. Environ. Sci. Technol..

[39] Perreault F., Tousley M.E., Elimelech M. (2014). Thin-Film composite polyamide membranes functionalized with biocidal graphene oxide nanosheets. Environ. Sci. Technol. Lett..

[40] Li M., Neoh K.G., Xu L.Q., Wang R., Kang E.T., Lau T., Olszyna D.P., Chiong E. (2012). Surface modification of silicone for biomedical applications requiring long-term antibacterial, antifouling, and hemocompatible properties. Langmuir.

[41] Huang J., Wang Z., Zhang J., Zhang X., Ma J., Wu Z. (2015). A novel composite conductive microfiltration membrane and its anti-fouling performance with an external electric field in membrane bioreactors. Sci. Rep..

[42] Zhang X., Wang Z., Tang C.Y., Ma J., Liu M., Ping M., Chen M., Wu Z. (2018). Modification of microfiltration membranes by alkoxysilane polycondensation induced quaternary ammonium compounds grafting for biofouling mitigation. J. Membr. Sci..

[43] Zhang F., Srinivasan M.P. (2004). Self-assembled molecular films of aminosilanes and their immobilization capacities. Langmuir.

[44] Liu D., Zhu J., Qiu M., He C. (2016). Antifouling PVDF membrane grafted with zwitterionic poly(lysine methacrylamide) brushes. RSC Adv..

[45] Banerjee I., Pangule R.C., Kane R.S. (2011). Antifouling coatings: Recent developments in the design of surfaces that prevent fouling by proteins, bacteria, and marine organisms. Adv. Mater..

[46] Liu C.X., Zhang D.R., He Y., Zhao X.S., Bai R. (2010). Modification of membrane surface for anti-biofouling performance: Effect of anti-adhesion and anti-bacteria approaches. J. Membr. Sci..

[47] Tiller J.C. (2010). Antimicrobial surfaces. Bioact. Surf..

[48] Kugler R., Bouloussa O., Rondelez F. (2005). Evidence of a charge-density threshold for optimum efficiency of biocidal cationic surfaces. Microbiology.

[49] Bieser A.M., Tiller J.C. (2011). Mechanistic considerations on contact-active antimicrobial surfaces with controlled functional group densities. Macromol. Biosci..

[50] Chen M., Zhang X., Wang Z., Liu M., Wang L., Wu Z. (2018). Impacts of quaternary ammonium compounds on membrane bioreactor performance: Acute and chronic responses of microorganisms. Water Res..

[51] Chen M., Zhang X., Wang Z., Wang L., Wu Z. (2017). QAC modified PVDF membranes: Antibiofouling performance, mechanisms, and effects on microbial communities in an MBR treating municipal wastewater. Water Res..

[52] Kaur R., Liu S. (2016). Antibacterial surface design-Contact kill. Prog. Surf. Sci..

[53] Murata H., Koepsel R.R., Matyjaszewski K., Russell A.J. (2007). Permanent, non-leaching antibacterial surfaces-2: How high density cationic surfaces kill bacterial cells. Biomaterials.

[54] Ferreira C., Pereira A.M., Pereira M.C., Melo L.F., Simoes M. (2011). Physiological changes induced by the quaternary ammonium compound benzyldimethyldodecylammonium chloride on Pseudomonas fluorescens. J. Antimicrob. Chemoth..

[55] Kolodkin-Gal I., Hazan R., Gaathon A., Carmeli S., Engelberg-Kulka H. (2007). A linear pentapeptide is a quorum-sensing factor required for mazEF-mediated cell death in Escherichia coli. Science.

