# Supplementary material for: Membrane Biofouling Control by Surface Modification of Quaternary Ammonium Compound Using Atom-Transfer Radical-Polymerization Method with Silica Nanoparticle as Interlayer
Source: Membranes (Basel). 2020 Dec 11;10(12):417. doi: 10.3390/membranes10120417 (PMC7764448; doi:10.3390/membranes10120417)
Supplement: Supplementary file 1 [file membranes-10-00417-s001.pdf]

membranes

Supplementary Materials

Membrane Biofouling Control by Surface Modification of Quaternary Ammonium Compound Using Atom-Transfer Radical-Polymerization Method with Silica Nanoparticle as Interlayer

Lehui Ren, Meng Ping and Xingran Zhang *

State Key Laboratory of Pollution Control and Resource Reuse, Shanghai Institute of Pollution Control and Ecological Security, School of Environmental Science and Engineering, Tongji University, 1239 Siping Road, Shanghai 200092, China; 1932834@tongji.edu.cn (L.R.); pingmeng@tongji.edu.cn (M.P.)

* Correspondence: xrzhang@tongji.edu.cn; Tel.: +86-21-65975669; Fax: +86-21-65980400

Contents

Additional details on materials and methods
Section S1. Membrane rejection behaviors.

Figures

Figure S1. The images of M0, MP, MSi, MBr and MQ.
Figure S2. Surface and cross-section morphologies of M0, MP, MSi, MBr and MQ.
Figure S3. Pore sizes of M0, MP, MSi, MBr and MQ.
Figure S4. CLSM 3D images of M0 and MQ membranes.

Section S1. Membrane rejection behaviors.

Rejection experiments were conducted with commercial SA as a model foulant representing polysaccharides in wastewater. SA rejection was tested at a TMP of 10 kPa using 100 mg/L of SA solution in a dead-end filtration cell. The concentrations in the feed and permeate solutions were determined in terms of total organic carbon (TOC, TOC-L, SHIMADZU, Japan). The rejection rate was calculated by the following equation.

$$R\,(\%) = \frac{C_\mathrm{f} - C_\mathrm{p}}{C_\mathrm{f}} \times 100 \quad (1)$$

where R is the rejection rate, C_f is the concentration of the feed solution (mg/L), and C_p is the concentration of the permeate solution (mg/L).

Figures

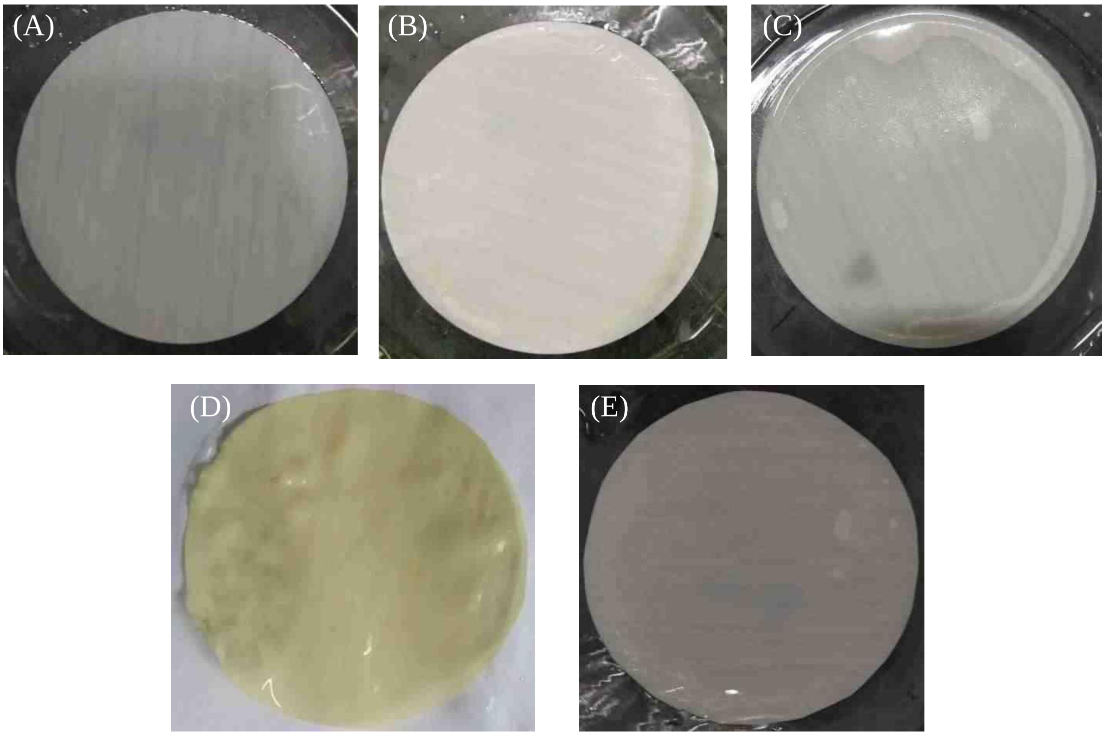

Figure S1. The images of (**A**) M0, (**B**) MP, (**C**) MSi, (**D**) MBr and (**E**) MQ.

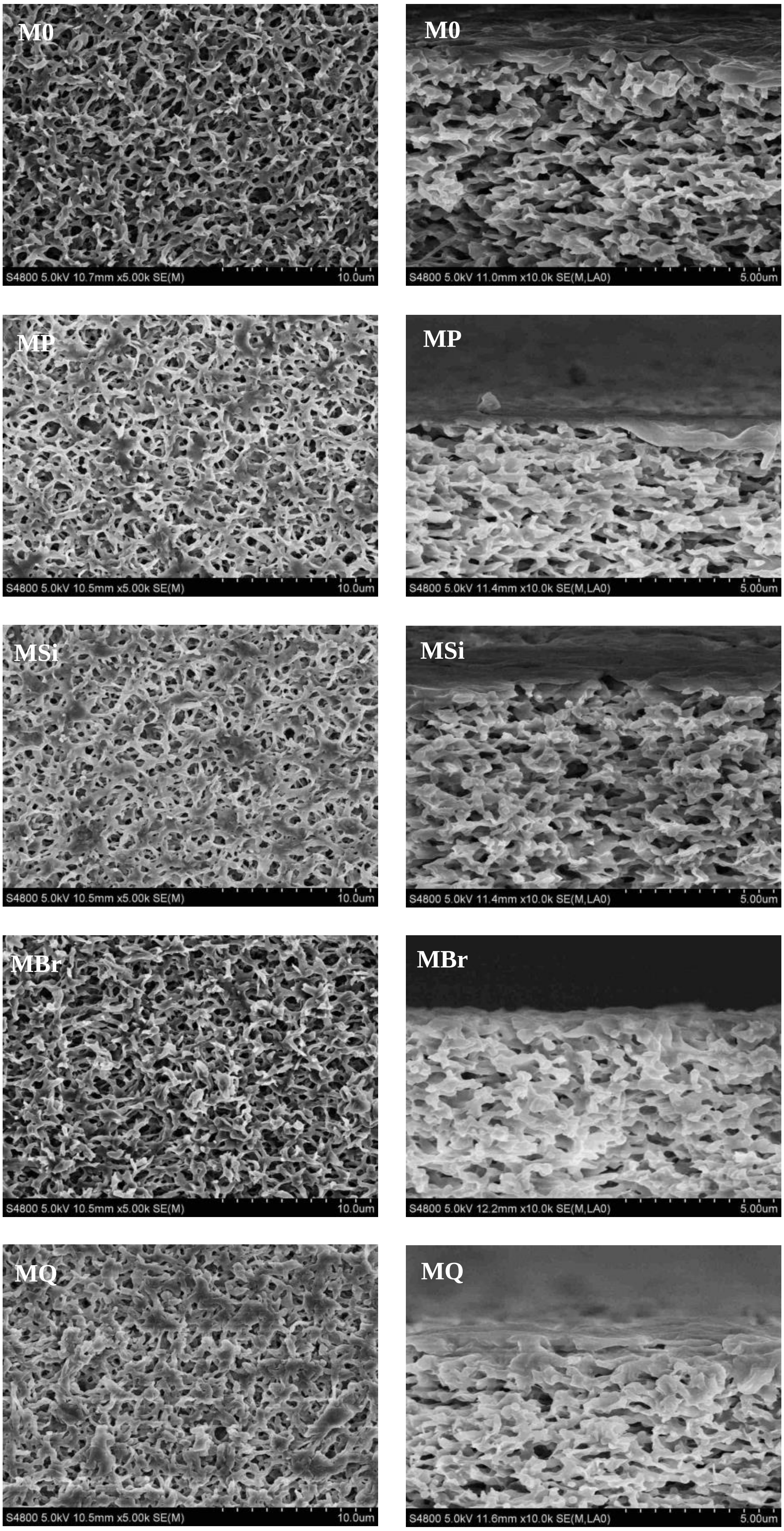

Figure S2. Surface and cross-section morphologies of M0, MP, MSi, MBr and MQ.

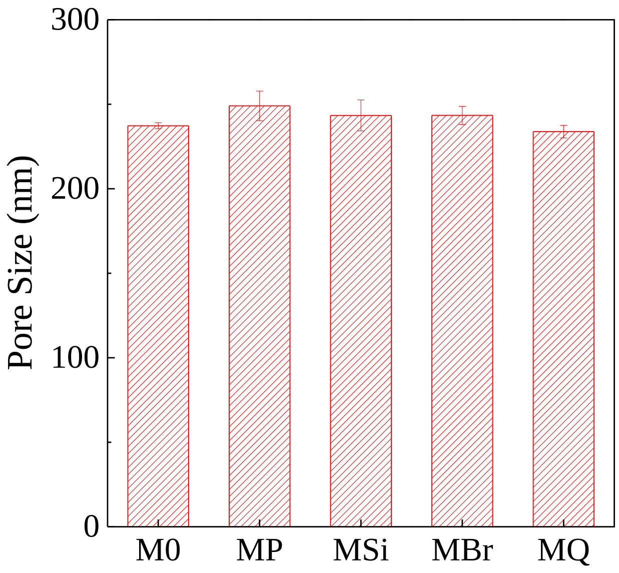

Figure S3. Pore sizes of M0, MP, MSi, MBr and MQ.

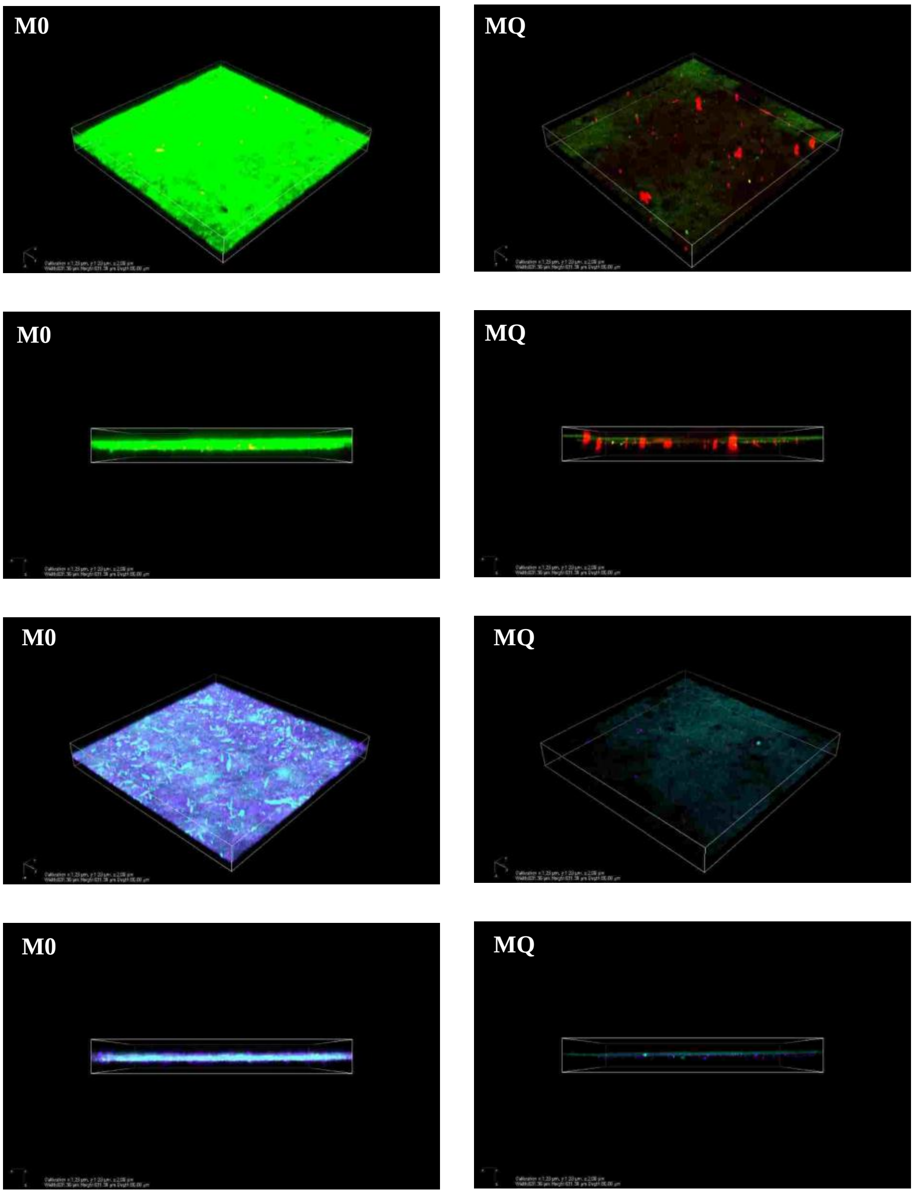

Figure S4. CLSM 3D images of M0 and MQ. SYTO 9 (green), PI (red), Con A (blue) and SYPRO Orange (yellow) were used to label "live", "dead" cells, α-polysaccharides and proteins in biofilms, respectively.
